# Supplementary material for: Impact of Hyponatremia on COVID-19-Related Outcomes: A Retrospective Analysis
Source: Biomedicines. 2024 Sep 2;12(9):1997. doi: 10.3390/biomedicines12091997 (PMC11444129; doi:10.3390/biomedicines12091997)
Supplement: Supplementary file 1 [file biomedicines-12-01997-s001.zip › biomedicines-3150828-supplementary.pdf]

Impact of sodium on COVID-19-related outcomes: a retrospective analysis

Pedro Maciel de Toledo Piza[1], Victor Muniz de Freitas[1], Isabella Aguiar-Brito[1], Barbara Monique Calsolari-Oliveira[1], Érika Bevilaqua Rangel[2,3*]

[1] Paulista School of Medicine, Federal University of São Paulo, São Paulo 04023-062, SP, Brazil

[2] Department of Medicine, Nephrology Division, Federal University of São Paulo, São Paulo 04038-031, SP, Brazil

[3] Instituto Israelita de Ensino e Pesquisa Albert Einstein, Hospital Israelita Albert Einstein, São Paulo 05652-900, SP, Brazil

Corresponding Author:

Name: Érika Bevilaqua Rangel, MD, PhD

Address: Nephrology Division, Department of Medicine, Federal University of São Paulo, Borges Lagoa Street, 591, 6th floor, Vila Clementino, São Paulo 04038-901, SP, Brazil.

Tel +55 11 5576-4848

e-mail: erikabr@uol.com.br

Table S1. Outcomes of patients admitted with COVID-19.

Outcome	Missing data (n, %)
Mortality	0
Intensive Care Unit	0
Oxygen (O_2)	2 (0.4)
Invasive Mechanical Ventilation	3 (0.5)
Renal Replacement Therapy	11 (2.0)

Table S2. Demographic data of patients admitted with COVID-19.

Demographic data	Missing data (n, %)
Age	0
Male	0
Obesity	185 (33.2)
Hypertension	184 (33)
COPD	184 (33)
Heart disease	184 (33)
Neoplasia	185 (33.2)
Respiratory disease	184 (33)
Liver disease	185 (33.2)
Transplant	184 (33)
Asthma	184 (33)
Diabetes mellitus	184 (33)
Cerebrovascular disease	184 (33)
Immunosuppressive therapy	184 (33)
Tuberculosis	184 (33)
Pregnancy	184 (33)
Smoking	184 (33)

COPD: chronic obstructive pulmonary disease.

Table S3. Signs and symptoms of patients admitted with COVID-19.

Sign and symptom	Missing data (n, %)
Fever	184 (33)
Fatigue	184 (33)
Sneezing	184 (33)
Dry cough	184 (33)
Productive cough	184 (33)
Rhinorrhea	184 (33)
Sore throat	184 (33)
Diarrhea	184 (33)
Dyspnea	184 (33)
Anorexia	184 (33)
Headache	184 (33)
Myalgia	185 (33.2)
Nausea/vomiting	185 (33.2)
Wheezing	184 (33)
Chest pain	185 (33.2)
Abdominal pain	184 (33)
Anosmia	184 (33)
Dysgeusia	185 (33.2)
Chills	184 (33)
Temperature	255 (45.7)
MBP	235 (42.1)
SBP	233 (41.8)
DBP	235 (42.1)
HR	233 (41.8)
SpO_2	220 (39.4)
Shock index	233 (41.8)
RR	249 (44.6)

MBP = mean blood pressure; SBP = systolic blood pressure; DBP = diastolic blood pressure; HR = heart rate (in bpm, beats per minute); SpO_2 = peripheral capillary oxygen saturation; RR = respiratory rate (in bpm, breaths per minute). Shock index is calculated as heart rate/systolic blood pressure.

Table S4. Laboratory parameters of patients admitted with COVID-19.

Laboratory parameter	Missing data (n, %)
Hemoglobin (g/dL)	5 (0.8)
Hematocrit (%)	8 (1.4)
Leucocytes (mm^3)	10 (1.8)
Neutrophils (mm^3)	9 (1.6)
Eosinophils (mm^3)	9 (1.6)
Basophils (mm^3)	8 (1.4)
Lymphocytes (mm^3)	9 (1.6)
Atypical lymphocytes (mm^3)	8 (1.4)
Monocytes (mm^3)	8 (1.4)
Platelets (x 10^3, mm^3)	16 (2.9)
Neutrophil-to-lymphocyte	10 (1.8)
Platelet-to-lymphocyte	16 (2.9)
D-dimer (µg/L)	160 (28.7)
Lactate (mmol/L)	121 (21.7)
CRP (mg/dL)	114 (20.4)
LDH (U/L)	217 (38.9)
Creatinine (mg/dL)	7 (1.3)
eGFR (mL/min/1,73m^2)	10 (1.8)
Urea (mg/dL)	7 (1.3)
Potassium (mEq/L)	13 (2.3)
Arterial blood glucose (mg/dL)	143 (25.7)
AST (U/L)	187 (33.5)
ALT (U/L)	165 (29.6)
pH	105 (18.9)
pCO_2 (mmHg)	105 (18.9)
pO_2 (mmHg)	107 (19.2)
Bicarbonate (mEq/L)	105 (18.9)
Base excess	107 (19.2)
ESR (mm)	525 (94.1)
Ferritin (ng/mL)	502 (90)
Albumin (g/dL)	483 (86.6)
Venous blood glucose (mg/dL)	448 (80.3)
γ-GT	410 (73.5)
Troponin (ng/L)	257 (46.1)

ESR = erythrocyte sedimentation rate; CRP = C-reactive protein; LDH = lactate dehydrogenase; eGFR = estimated glomerular filtration rate; AST = aspartate aminotransferase; ALT = alanine aminotransferase; γ-GT = gamma-glutamyl transferase.
